# Variable Ki67 proliferative index in 65 cases of nodular fasciitis, compared with fibrosarcoma and fibromatosis

**DOI:** 10.1186/1746-1596-8-50

**Published:** 2013-03-26

**Authors:** Xu-Yong Lin, Liang Wang, Yong Zhang, Shun-Dong Dai, En-Hua Wang

**Affiliations:** 1 Department of Pathology, the First Affiliated Hospital and College of Basic Medical Sciences, China Medical University, Shenyang 110001, China; 2 Institute of pathology and pathophysiology, China Medical University, Shenyang 110001, China

**Keywords:** Nodular fasciitis, Ki67, Desmoid fibromatosis, Fibrosarcoma

## Abstract

**Abstract:**

Nodular fasciitis is the most common pseudosarcomatous lesion of soft tissue. Ki67 was considered as a useful marker for distinguishing some benign and malignant lesions. To study the usefulness of Ki67 in diagnosis of nodular fasciitis, the expression of Ki67 was examined by using immunostaining in 65 nodular fasciitis specimens, 15 desmoid fibromatosis specimens and 20 fibrosarcoma specimens. The results showed that there was a variable Ki67 index in all 65 cases of nodular fasciitis, and the mean labeling index was 23.71±15.01%. In majority (70.77%) of all cases,the index was ranged from 10% to 50%, in 6.15% (4/65) of cases the higher Ki67 index (over 50%) could be seen. The Ki67 proliferative index was closely related to duration of lesion, but not to age distribution, lesion size, sites of lesions and gender. Moreover, the mean proliferative index in desmoid fibromatosis and fibrosarcoma was 3.20±1.26% and 26.15±3.30% respectively. The mean Ki67 index of nodular fasciitis was not significantly lower than fibrosarcoma, but higher than desmoid fibromatosis. The variable and high Ki67 index in nodular fasciitis may pose a diagnostic challenge. We should not misdiagnose nodular fasciitis as a sarcoma because of its high Ki67 index. The recurrence of nodular fasciitis is rare; and the utility of Ki67 immunostaining may be not suitable for recurrence assessment in nodular fasciitis.

****Virtual slides**:**

The virtual slide(s) for this article can be found here: http://www.diagnosticpathology.diagnomx.eu/vs/4782335818876666

## Background

Nodular fasciitis(NF) is the most common pseudosarcomatous lesion of soft tissue. This lesion was often misdiagnosed as a sarcoma because of its rapid growth, rich cellularity, and mitotic activity [1,2]. Recently USP6 rearrangement and MYH9-USP6 gene fusions have been identified in a high percentage of NF suggestive that this is the driving translocation [3]. Histologically, NF is mainly made up of plump myofibroblasts which resemble the fibroblasts in tissue culture or granulation tissue, and the background is variably myxoid or fibrous [4]. The correct diagnosis is usually based on the history of rapid growth, lesion size and the histological appearance. Even so, sometimes it is still hard to distinguish NF from fibrosarcoma or myxoid fibrosarcoma. So, additional diagnostic markers may be helpful to distinguish between these lesions. Ki67 antigen is a cell cycle–associated nuclear antigen and is present in all stages of the cell cycle (G1,S,G2, and M phases) in proliferating cells but is absent in G0 and early G1phases of cells re-entering the cell cycle [5]. The monoclonal antibody of Ki67 protein, MIB1, has been documented useful for the diagnosis [6-10] and prognosis [11,12] of some neoplasms. So far, in addition to Ki67 expression in 3 cases of nodular fasciitis described by Ooe M [13], there is no wide and deep report about the expression of Ki67 in NF. To further study the usefulness of Ki67 in diagnosis of NF, we selected 65 cases of NF, 15 cases of desmoid fibromatosis and 20 cases of fibrosarcoma, and examined the expression of Ki67 in them by immunohistochemical method.

## Materials and methods

### Patients and specimens

We collected 65 cases of NF from the First Affiliated Hospital of China Medical University from 1995 to 2009. The patients with NF included 37 men and 28 women. Ages of all patients ranged from 8 to 84 years, and the average age was 37 years. The clinical findings were summarized in Table 1. Postoperative follow up was obtained in 45 patients and ranged from 12 to 66 months (mean follow up, 31 months). In addition, 15 cases of desmoid fibromatosis and 20 cases of fibrosarcoma were selected as controls. All specimens were re-evaluated for diagnosis following the criteria for classification of soft tissue tumor by the World Health Organization (WHO). This study was conducted according to the regulations of the institutional review boards (China Medical University).

**Table 1:** The sites, lesion size, duration and age distribution in 65 cases of nodular fasciitis

**Characteristic**	**N**	**Rate**
Age (years)		
<10	3	4.62%
10-19	7	10.77%
20-29	13	20.00%
30-39	21	32.31%
40-60	16	24.62%
>60	5	7.69%
Size (cm)		
≤1	7	10.77%
>1, ≤2	23	35.38%
>2, ≤3	20	30.77%
>3	15	23.08%
Duration (months)		
≤1	11	16.92%
>1, ≤2	15	23.08%
>2, ≤3	16	24.62%
>3, ≤5	19	29.23%
>5	4	4.62%
Sites		
upper extremity	25	38.46%
trunk	18	27.69%
head and neck	10	15.38%
Lower extremity	9	13.85%
others	3	4.62%
Ki67 labeling index (%)		
≤10	15	23.08%
10—30	32	49.23%
31—50	14	21.54%
>51	4	6.15%

### Immunohistochemical staining

All the resected specimens were fixed with 10% neutral-buffered formalin and embedded in paraffin blocks. Tissue blocks were cut into 4-μm slides, deparaffinized in xylene, rehydrated with graded alcohols, and immunostained with anti-Ki67(MIB-1, mouse monoclonal antibody, Santa, USA). In addition, S-100, CD34, Desmin, cytokeratin (CK), Vimentin and α-SMA were also used for making the diagnosis.

Sections were stained with a streptavidin-peroxidase system (KIT-9720, Ultrasensitive TM S-P, MaiXin, China). The chromogen used was diaminobenzidine tetrahydrochloride substrate (DAB kit, MaiXin, China), slightly counterstained with hematoxylin, dehydrated and mounted. For the negative controls, the primary antibody was replaced with PBS. The sections were observed under five high power fields which showed the relatively higher Ki67 staining, and 500 tumor cells (exclusive of red cells and inflammatory cells) were counted in each field. The location of Ki67 staining was on the nuclear.

### Statistical analysis

All statistical calculations were performed by SPSS version 18.0 for Windows software (SPSS Inc, IL, USA). The chi-square test or the *t* test was used to compare data from Ki67 staining. *P* values less than 0.05 were considered statistically significant.

## Results

### Clinical findings and histological features

Of all 65 patients with NF, the majority of them suffered the rapid growth mass, and 30.77% (20/65) of patients had the tenderness or slight pain. The sites, lesion size, duration and age distribution were listed in Table 1.

**Figure 1:**
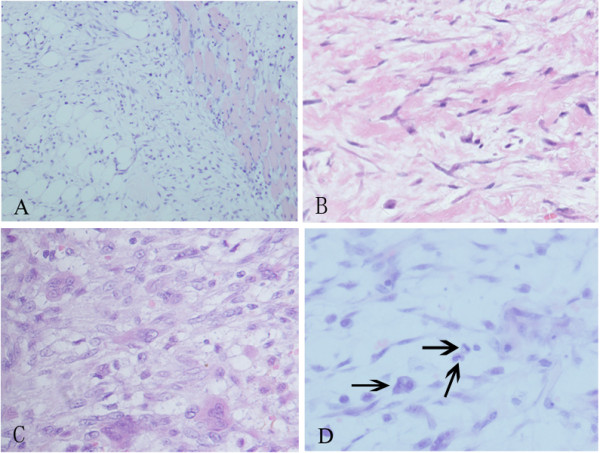
**Morphological changes of nodular fasciitis.** (**A**) Spindle cells were diffused in extensive myxoid stroma. At the margin of the tumor, the cells infiltrated the normal skeletal muscle tissue. (**B**) Focally, keloid like collagen bundles present in the peripheral area. (**C**) Extravasated erythrocytes, scattered osteoclast-like giant cells were sprinkled between the plump myofibroblasts (**D**) Numerous mitoses (black arrow) were present.

Histologically, all cases showed variably myxoid to fibrous, which composed of plump immature-appearing myofibroblasts. The cells had oval, pale-staining nuclei with prominent nucleoli. In some cases, highly cellular areas could be seen, and the myofibroblasts showed plentiful mitotic figures (Figure 1).

### Imunohistochemical staining

**Figure 2:**
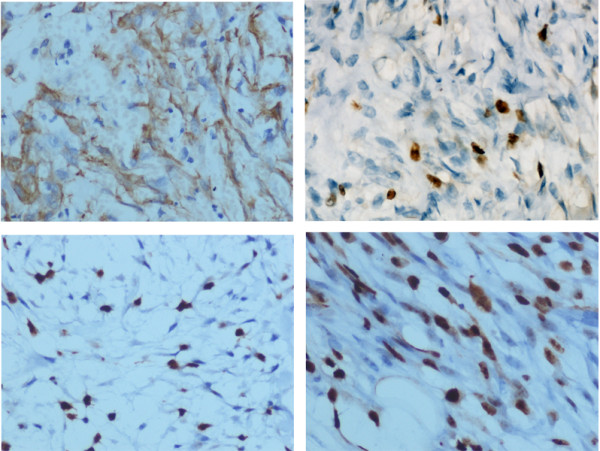
**Immunohistochemical staining of α-SMA, Ki67 in nodular fasciitis.** (**A**) Nodular fasciitis showed a diffuse expression forα-SMA; (**B**) The Ki67 index was approximately 5%; (**C**) The Ki67 index was approximately 20%; (**D**) The Ki67 index was approximately 50%.

By imunohistochemical staining, all cases of NF were positive for Vimentin and SMA, negative for S-100, CD34, Desmin and CK, and Ki67 was expressed in all 65 NF specimens. In 23.08% (15/65) of all cases, the Ki67 labeling index was less than 10%, while in 6.15% (4/65) Ki67 index was over 50% (Figure 2). The majority (70.77%) of them were ranged from 10–50% (Table 1). The mean index was 23.71±15.01%.

The mean Ki67 index in 15 cases of desmoid fibromatosis was 3.20±1.26%, significantly lower than that in NF (*P*<0.0005). The mean Ki67 index in 20 cases of fibrosarcoma was 26.15±3.30%, not significantly higher than that in NF (*P* =0.472) (Table 2).

**Table 2:** The Ki67 index in nodular fasciitis, desmoid fibrosatois and fibrosarcoma

**Lesions**	**N**	**Mean±SD**	***P***
Nodular fasiitis	65	23.71±14.05	
Fibromatosis	15	3.20±1.26%	0.000
Fibrosarcoma	20	26.15±3.30%	0.472

There was no significant difference in different age distribution, lesion size, sites of lesions and gender, while the significant difference was found in different duration of lesion (*P* =0.022). That is, the Ki67 index in cases of NF with the duration no more than 2 months was significantly higher than these with the duration more than 2 months (Table 3). In contrast, the Ki67 index in fibrosarcoma and fibromatosis was not associated with the duration (Table 4).

**Table 3:** The expression of Ki67 in 65 cases of nodular fasciitis with different age distribution, lesion size, sites of lesions and gender

**Characteristic**	**N**	**Rate**	**Mean±SD**	***P***
Age (years)				
<20	10	15.38	25.30±15.97	0.932
20-39	34	52.31	23.24±15.71	
>40	21	32.31	23.71±14.05	
Size (cm)				
≤2	30	46.15	27.03±14.75	0.097
>2	35	53.85	20.86±14.70	
Duration (months)				
≤2	26	40.00	28.23±15.88	0.022
>2	39	60.00	19.80±13.11	
Sites				
Upper extremity	25	38.46	23.28±15.51	0.964
Trunk	18	27.69	24.06±13.82	
Head and neck	10	15.38	23.00±16.49	
Lower extremity	9	13.85	22.78±14.37	
Others	3	4.62	30.00±20.91	
Gender				
Male	37	56.92	22.50±16.07	0.102
Female	28	43.08	16.67±12.28	

**Table 4:** The relationship between Ki67 index and duration in nodular fasciitis, fibrosarcoma and fibromatosis

**Lesion**	**Duration (months)**	**N**	**Rate**	**Mean±SD**	***P***
Nodular fasciitis	≤2	26	40.00	28.23±15.88	0.022
	>2	39	60.00	19.80±13.11	
Fibrosarcoma	≤2	7	35.00	26.57±3.78	0.474
	>2	13	65.00	25.92±3.15	
Fibromatosis	≤2	4	26.67	3.50±1.29	0.953
	>2	11	73.33	3.09±1.30	

### Follow up

Before the end of follow up, two patients (2/45, 4.44%) found a recurrence 18 and 26 months after the resection respectively. One of the recurred cases was from head and neck, another was from lower extremity. The duration was 5 months and 6 months respectively. And the Ki67 index in the two recurred cases was approximately 9% and 15% respectively.

## Discussion

NF is a pseudosarcomatous, self-limiting reactive process which was first described by Kornwaler et al. in 1955 as “subcutaneous pseudosarcomatous fibromatosis” [14]. Histologically, NF consists mainly of plump immature fibroblasts that are arranged in short irregular bundles and fascicles, accompanied by a dense reticulin meshwork with small amounts of mature collagen. Extravasated erythrocytes, scattered lymphocytes and osteoclast-like giant cells are often sprinkled between the myofibroblasts. Although mitotic figures are fairly common, atypical mitosis is virtually never seen [2,15]. Thus, the correct diagnosis of NF mainly depends on tumor size, short duration, red blood cell extravasation, keloid-type collagen, and lack of markedly cellular atypia. In contrast, fibrosarcoma typically occurs in patients older than 50 years and measures more than 3 cm. The cells show more nuclear pleomorphism, atypical mitotic figures. Fibromatosis consists of bland cells arranged in sweeping bundles with dense collagenous stroma and usually lacking inflammatory component, thin-walled vessels and extravasated red blood cells.

Despite this entity has been well described, NF is still often misdiagnosed as a sarcoma or other malignancies because of its rapid growth, rich cellularity, and mitotic activity. So, in addition to histological appearances, some diagnostic markers including proliferative markers should be used for differential diagnosis. Ki67 antigen is a marker of proliferating cells, and is rapidly degraded after mitosis. For the short half-time, it has been suggested that Ki67 staining is more accurate and specific than the counting of mitoses or PCNA staining [16]. Oshiro Y et al. ever reported that NF has higher expression of proliferating cell nuclear antigen than other benign and malignant fibroblastic lesions [17]. However, the utility of the proliferative markers has not been established for distinguishing between these lesions for diagnostic purpose [18]. So far, in addition to Ki67 expression in 3 cases of NF described by Ooe M [13], there is no deep study on the expression of Ki67 in NF. So, to further detect the diagnostic usefulness of Ki67, we examined the expression of Ki67 in 65 cases of NF, 15 cases of desmoid fibromatosis and 20 cases of fibrosarcoma. The imunohistochemical staining results showed although Ki67 labeling index was variable in NF, there was generally a higher expression level (mean index 23.71±15.01%). Ki67 has been proved to be a useful marker for distinguishing many benign and malignant lesions [6,7]. Gong et al. [19] found a significantly higher expression of Ki67 in ghost cell odontogenic carcinoma than in calcifying cystic odontogenic tumor. But, our results revealed that NF could show the higher expression of Ki67. The mean Ki67 index in 15 cases of desmoid fibrosatois is significantly lower than that in nodular fasiitis, however, the mean Ki67 index in 20 cases of fibrosarcoma is not significantly higher than that in NF. This result indicates that usefulness of Ki67 staining in differential diagnosis of NF is limited, and may represent a diagnostic pitfall. And we should not misdiagnose the lesion as a sarcoma because of its higher proliferative index. The higher proliferative index may further indicate it is a lesion which closely mimics a sarcoma. However, in contrast to the average index 5.6 reported by Ooe M et al. [13], our Ki67 index is much higher. It may be because that the numbers of specimens selected by Ooe M et al. is too small, or the counting method is different.

In addition, we found that the Ki67 labeling index was closely related to duration of lesion, but not to age distribution, lesion size, sites of lesions and gender. This suggests that if the duration was shorter, the myofibroblasts might have the higher proliferative activities, so the cells showed the higher expression of Ki67. That is, in the earlier stage of the lesion, the majority of myofibroblasts might be in immature status, conversely, if the lesion had a long history, only few of the proliferative cells might have high proliferative activities. In contrast, the Ki67 index in fibrosarcoma and fibromatosis was not associated with the duration. This phenomenon may represent self-limiting reactive process of NF. Acording to Allen, nearly all nodules have been effectively treated by local excision, in the series of 895 cases only 9 (1%) reappeared after attempted complete surgical excision [20]. Thompson and colleagues found a local recurrence rate of 9.3% in cases of NF of the external ear, a much higher rate of local recurrence when compared to NF at other sites [21]. Our follow up results reveal a 4.44% recurrence rate, generally similar to the reported previously, indicating the recurrence of NF is rare. Surprisingly, the Ki67 index in two recurred cases was approximately 9% and 15% respectively, relatively not so high. In contrast, Hoos et al. [22] and Hase-gawa et al. [23] have reported that analysis of Ki67 can identify tumors with aggressive behavior also among tumors with other high-risk characteristics. That is, the higher Ki-67 index usually means the poorer prognosis and the higher recurrence rate. But our finding indicated that the Ki67 staining may be not suitable for recurrence assessment in NF.

## Conclusion

Our results indicate there is a variable expression in all 65 cases of NF. The variable expression of Ki67 in NF may cause a diagnostic confusion. We should not misdiagnose NF as a sarcoma because of its high Ki67 expression. The Ki67 proliferative index may be related to the duration of the leison. The Ki67 staining may be not suitable for prognosis assessment in NF.

## Consent

Written informed consent was obtained from the patient for publication of this case report and accompanying images. A copy of the written consent is available for review by the Editor-in Chief of this Journal.

## Competing interests

The authors declare that they have no competing financial interests. No part of this article has been published or submitted elsewhere, and there are no financial or other relationships that might lead to a conflict of interest of this article.

## Authors’ contributions

LXY and WL participated in the histopathological evaluation, performed the literature review, acquired photomicrographs and drafted the manuscript. ZY carried out the immunohistochemical stains evaluation. DSD conceived and designed the study. WEH revised the manuscript. All the authors read and approved the final manuscript.
